# Targeting NAD+ in Metabolic Disease: New Insights Into an Old Molecule

**DOI:** 10.1210/js.2017-00092

**Published:** 2017-05-15

**Authors:** Yasir S. Elhassan, Andrew A. Philp, Gareth G. Lavery

**Affiliations:** 1 Institute of Metabolism and Systems Research, University of Birmingham, Birmingham B15 2TT, United Kingdom; 2 Centre for Endocrinology, Diabetes and Metabolism, Birmingham Health Partners, Birmingham B15 2TT, United Kingdom; 3 MRC-ARUK Centre for Musculoskeletal Ageing Research, School of Sport, Exercise and Rehabilitation Sciences, University of Birmingham, Birmingham B15 2TT, United Kingdom

**Keywords:** nicotinamide riboside, nicotinamide mononucleotide, mitochondria, diabetes, nonalcoholic fatty liver disease, aging

## Abstract

Nicotinamide adenine dinucleotide (NAD+) is an established cofactor for enzymes serving cellular metabolic reactions. More recent research identified NAD+ as a signaling molecule and substrate for sirtuins and poly-adenosine 5′-diphosphate polymerases; enzymes that regulate protein deacetylation and DNA repair, and translate changes in energy status into metabolic adaptations. Deranged NAD+ homeostasis and concurrent alterations in mitochondrial function are intrinsic in metabolic disorders, such as type 2 diabetes, nonalcoholic fatty liver, and age-related diseases. Contemporary NAD+ precursors show promise as nutraceuticals to restore target tissue NAD+ and have demonstrated the ability to improve mitochondrial function and sirtuin-dependent signaling. This review discusses the accumulating evidence for targeting NAD+ metabolism in metabolic disease, maps the different strategies for NAD+ boosting, and addresses the challenges and open questions in the field. The health potential of targeting NAD+ homeostasis will inform clinical study design to identify nutraceutical approaches for combating metabolic disease and the unwanted effects of aging.

## Teaser

This review covers recent developments in NAD+ biology and the advent of nutraceutical molecules that inform on the pathophysiology of metabolic disorders, potentially illuminating areas of therapeutic benefit.

Nicotinamide adenine dinucleotide (NAD+) was discovered more than 100 years ago by Sir Arthur Harden as a low-molecular-weight substance present in boiled yeast extracts [1]. In the late 1920s, Joseph Goldberger fed Brewer’s yeast to dogs with pellagra, a devastating disease characterized by dermatitis, diarrhea, dementia, and death, and their health improved. At that time, pellagra was endemic in parts of the United States, and so the Red Cross supplemented Brewer’s yeast to its food rations in pellagra-endemic areas; within weeks the disease burden dissipated [2, 3]. The health significance of NAD+ was established in 1937, when Conrad Elvehjem and his colleagues made the major discovery that the factor that prevented and cured pellagra was the NAD+ precursor, nicotinic acid [4, 5].

NAD+ plays a central role in cellular respiration, the cascade of reactions that generate adenosine triphosphate (ATP) from nutrient breakdown, by acting as a coenzyme for oxidoreductases and dehydrogenases [6–9]. As coenzymes, NAD+ and its phosphorylated and reduced forms, including NADP+, NADH, and NADPH, are critical for the activities of cellular metabolism and energy production [1, 10, 11]. NAD+ most commonly functions in energy-generating catabolic reactions (such as glycolysis, fatty oxidation, and citric acid cycle), where it is reduced to NADH, which is then shuttled into the mitochondria to generate ATP. This generates an NAD+/NADH ratio, which is useful to assess the health and energy charge of the cell. The phosphorylated form, NADP(H)+, participates in anabolic reactions, such as fatty acid and cholesterol synthesis [8, 9, 12].

**Figure 1.**
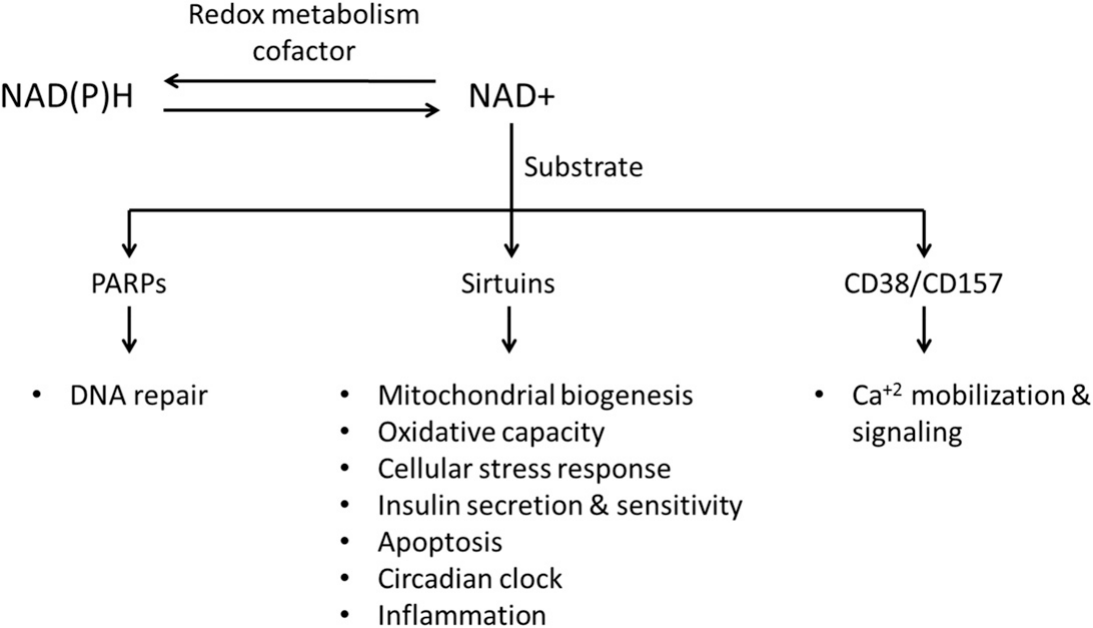
NAD+ as a redox cofactor and a consumed substrate.

More recently and as importantly, NAD+ has been studied as a rate-limiting substrate for three classes of enzymatic reactions involved in posttranslational modification (Fig. 1), all of which exhibit breaking of the glycoside bond between nicotinamide and the adenosine 5′-diphosphate (ADP)-ribose moiety, and the latter is then transferred onto an acceptor molecule [6–9, 11]. The first class includes mono- and poly-ADP ribose transferases, among which the poly-ADP ribose polymerases (PARPs) are the most studied and are classically described as DNA repair proteins [13, 14]. The second class is the cyclic ADP ribose synthases (CD38 and CD157), which are membrane-bound ectoenzymes that produce and hydrolyze the Ca2+-mobilizing second messenger cyclic ADP-ribose from NAD+ and are therefore key in calcium homeostasis and signaling [15]. The third and most important class in terms of cellular energy metabolism consists of the sirtuins, named for their similarity to the yeast Sir2 gene-silencing protein. Seven sirtuins exist in mammals (SIRT1 through SIRT7), with diverse enzymatic activities, expression patterns, cellular localizations, and biological functions [16]. Sirtuins have a host of metabolic targets, resulting in profound effects on various cellular processes, such as mitochondrial biogenesis, cellular stress response, lipid metabolism, insulin secretion and sensitivity, apoptosis, circadian clock dynamics, inflammation, and aging [17]. Through these targets, sirtuins translate changes in feeding status, DNA damage, and oxidative stress into metabolic adaptations [18–20]. SIRT1, the most-studied sirtuin, targets multiple transcriptional coactivators, such as the peroxisome proliferator-activated receptor *γ*coactivator-1*α* (PGC-1*α*) and transcription factors, such as the forkhead box protein O1. PGC-1*α* is a central regulator of energy metabolism and mitochondrial biogenesis [21–24], whereas forkhead box protein O1 regulates mitochondrial fatty acid metabolism and protects against oxidative stress [25–27]. As nutrients influence the NAD+/NADH pool, these NAD+-dependent signaling reactions are recognized as the sensors of metabolism owing to their decisive regulatory roles in cellular metabolism [17]. Appropriate regulation of these NAD+-dependent processes relies on the cellular ability to conserve their NAD+ content. Therefore, inadequate NAD+ homeostasis can be pathologic, linked to impaired cell signaling and mitochondrial function [19, 28, 29].

The dependency of sirtuins on NAD+ [30], and the finding that yeast Sir2 protein is required for the lifespan extension mediated by caloric restriction (CR) [31], led to a renascent interest in NAD+ metabolism research, centered on modifying NAD+ availability to support sirtuin-mediated cellular metabolism to mimic CR. This interest was enhanced by the discovery of contemporary NAD+ precursors that can circumvent issues with existing molecules, which can also increase NAD+ *in vivo* and human tissues [32–34]. As we review here, these key findings underline the prospect of targeting NAD+ biosynthetic pathways to increase mitochondrial function and sirtuin activity in the combat against metabolic disease. We also highlight the challenges and the knowledge gaps that require investigating before these compounds can find their way to the clinics.

## 1. NAD+ Biosynthesis and Metabolism

**Figure 2.**
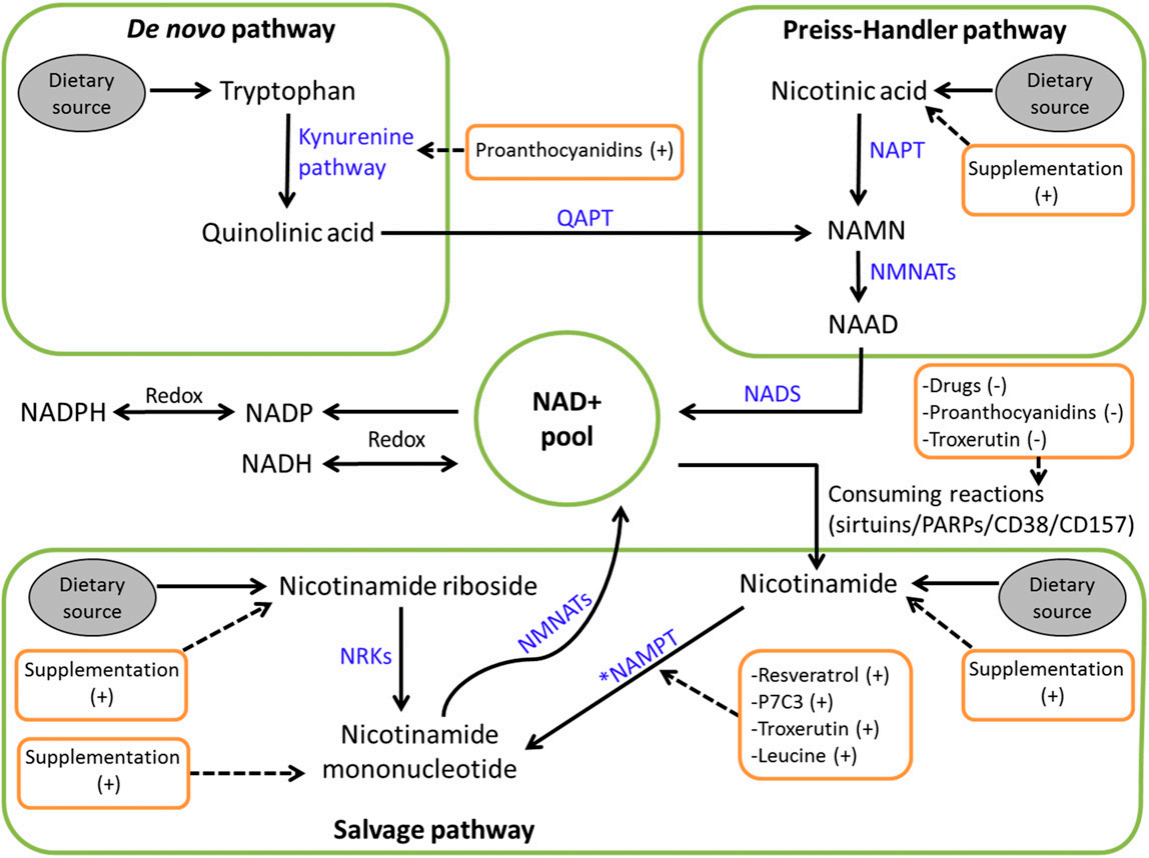
Schematic overview of human NAD+ biosynthesis. NAAD, nicotinic acid adenine dinucleotide; NADS, NAD+ synthase; NAPT, nicotinic acid phosphoribosyltransferase; NMNAT, nicotinamide mononucleotide adenylyltransferase; QAPT, quinolinic acid phosphoribosyltransferase. *NAMPT is the rate limiting step in NAD+ biosynthesis.

In humans, NAD+ can be synthesized via the *de novo/*kynurenine pathway from the amino acid tryptophan [35, 36]. However, tryptophan is a poor NAD+ precursor *in vivo* [37]. Most organisms have alternative NAD+ synthesis pathways (Fig. 2) from the dietary vitamin B3 precursors nicotinic acid (NA), nicotinamide (Nam), and nicotinamide riboside (NR), or from a salvage pathway where the Nam molecule split from NAD+-consuming reactions is recycled into nicotinamide mononucleotide (NMN) via the rate-limiting enzyme nicotinamide phosphoribosyltransferase (NAMPT), and NAD+ is regenerated [9, 11, 35, 38–41]. In addition, a more recently described salvage pathway recycles NR to NMN via the nicotinamide riboside kinases (NRKs) [32]. In humans, these different routes to NAD+ synthesis converge at the NAD+ and nicotinic acid adenine dinucleotide formation step catalyzed by the nicotinamide mononucleotide adenylyltransferases. Nicotinic acid adenine dinucleotide is then amidated to form NAD+.

Nicotinic acid riboside is an NAD+ biosynthesis intermediate that can be converted in yeast and human cells by NRKs into nicotinic acid mononucleotide and then to NAD+ [42]. It is the least-studied NAD+ precursor and is therefore beyond the scope of this review.

The energy sensor adenosine monophosphate-activated protein kinase (AMPK), which adapts cells to low-energy states in the support of ATP production [43, 44], activates NAMPT, increases NAD+ recycling, and enhances SIRT1 activity [45, 46].

In mammals, the entire NAD+ pool is used and replenished several times a day, balanced by the distinct NAD+ biosynthetic pathways [47]. Owing to its constant utilization, the half-life of NAD+ in mammals is short (up to 10 hours) [36, 48–51], with intracellular levels believed to be 0.4 to 0.7 mM [41]; however, the accuracy of this level depends on the cell type and physiologic state being assessed. It is clear that NAD+ concentrations differ substantially between cellular compartments, with mitochondrial NAD+ concentration being the highest and representing 70% to 75% of cellular NAD+ (10- to 100-fold higher than those in the cytosol) [52, 53]. The NAD+/NADH levels vary to adjust cellular and tissue physiology in response to changes in nutrient availability and energy demand. For instance, NAD+ levels drop in response to high-fat diet (HFD) in mice [33, 54] and with aging, contributing to age-related disorders, such as diabetes, cardiovascular disease, cancer, and neurodegenerative disease [55–58]. Conversely, the renowned health adaptive beneficial effects of CR and exercise have been linked to NAMPT activation and the subsequent rise in NAD+, sirtuins, and mitochondrial activity [46, 59–61].

## 2. Therapeutic NAD+ Boosting

The recommended daily allowance (RDA) of niacin, a collective term for NA and Nam, is around 15 mg/d and can be met through the consumption of meat, fish, and dairy products [12, 62]. More recently, NR was also detected in milk and yeast [32, 63].

A plethora of evidence suggests that higher rates of NAD+ synthesis can positively affect pathways that require NAD+ as a cosubstrate. The NAD+ pools can be elevated via provision of precursors [33, 54, 64, 65], NAD+ biosynthesis augmentation [45, 46], and inhibition of NAD+ consumers [57, 66–68].

## 3. NAD+ Precursor Supplementation

The most tractable approach to increase NAD+ would be via the supplementation of the different precursors, all of which increase NAD+ levels in human and animal tissues. This approach is the focus of this review because NAD+ precursors are naturally occurring in food and are readily available in isolated forms, allowing nutritional approaches to be applied to modulate NAD+ metabolism *in vivo*.

### A. Niacin

NA has been used for >50 years in the treatment of hyperlipidemia [69, 70]. Dietary niacin is not associated with side effects because the tolerable upper intake level is not exceeded [62], whereas pharmacologic NA dosing is commonly associated with undesirable effects, thereby decreasing treatment adherence. NA is a ligand for the G-protein–coupled receptor GPR109A and is coexpressed on the epidermal Langerhans cells mediating prostaglandin formation, which induces troublesome flushing and other vasodilatory effects, such as itching, hypotension, and headaches [12, 71–73]. To overcome these problems, the selective antagonist of prostaglandin D2 receptors, laropiprant, was introduced into clinical practice in combination with extended-release NA (extended-release NA-laropiprant) [74]. Extended-release NA-laropiprant failed to prove advantageous in clinical trials; safety concerns arose, and the agent was therefore withdrawn from all markets [75]. A long-acting NA analog, acipimox, is undergoing clinical trials [76–79]. However, acipimox remains a GPR109A receptor ligand [80], thus retaining the potential for undesirable side effects that will limit its clinical utility.

Although Nam is the predominant endogenous precursor of the NAD+ salvage pathway, early reports suggested that it may not be as effective as other biosynthesis precursors in increasing NAD+ levels [41]; however, this likely reflects the relatively small dose of Nam used. Additionally, Nam effects likely depend on cell/tissue type and the pathophysiologic state. For instance, in a nonstressed state, Nam is inferior to NA as an NAD+ precursor in the liver [81], whereas under HFD-induced metabolic challenge, Nam is a more powerful NAD+ precursor and SIRT1 activator than NA [82]. Nam has been used for many years for a variety of therapeutic applications (such as diabetes mellitus) at doses up to 3 g/d, with minimal side effects [83]. Unlike NA, Nam has no GPR109A agonist activity [80], thus escaping the prostaglandin-mediated vasodilatory side effects. Yet, at high doses Nam has a toxic potential (particularly hepatotoxicity), raising health concerns [83] and, as well as with long-term use, can cause negative feedback to inhibit sirtuins [84, 85].

### B. NR and NMN

NR has been recognized since the 1950s as an NAD+ precursor in bacteria that lack the enzymes of the *de novo* and Preiss–Handler pathways [86–88]. This changed in 2004, when Bieganowski and Brenner [32] detected the presence of NR in milk and identified two human NRK enzymes capable of synthesizing NAD+ from NR. Subsequent human and animal studies confirmed that NR can increase intracellular NAD+ in a dose-dependent fashion [34, 89, 90]. Likewise, NMN is an intermediate in the NAD+ salvage pathway. Although less studied than NR, several studies proved that NMN increases NAD+ levels *in vitro* and *in vivo* [33, 56, 91–93]. Several recent studies using NR and NMN have attracted major research interest and are discussed later.

## 4. NAD+ Biosynthesis Augmentation

Several AMPK and NAMPT activators have been studied. Resveratrol is a nonflavonoid polyphenol that is present in red grapes, wine, and pomegranates; activates AMPK and SIRT1; and improves metabolic health status in humans [94–98]. However, conflicting outcomes from clinical studies have questioned the efficacy of resveratrol in treating human metabolic disease [99]. Nonetheless, it remains a compound of substantial interest to many [100].

Various AMPK activators exist [101]. Among them is metformin, which was introduced in the 1950s to treat diabetes, with a multitude of favorable metabolic outcomes that rely on AMPK [102]. Cantó *et al.* [45] reported that the AMPK activators metformin and 5-aminoimidazole-4-carboxamide ribonucleotide, increase NAD+ and sirtuin activity, thereby regulating energy expenditure .

Other compounds have also been reported to increase NAMPT activity. P7C3, a neuroprotective chemical that enhances neuron formation, can bind NAMPT and increase NAD+ levels [103–105]. Likewise, the antioxidant troxerutin, a trihydroxyethylated derivative of the natural bioflavonoid rutin, markedly increased NAD+ levels and potentiated SIRT1 via NAMPT activation and PARP1 inhibition in HFD-treated mouse liver [106]. Remarkably, leucine supplementation in obese mice also increased *NAMPT* expression and enhanced intracellular NAD+ levels [107]. Moreover, proanthocyanidins, the most abundant flavonoid polyphenols in human diet, can dose-dependently increase NAD+ levels in rat liver via the increased expression of the *de novo* pathway enzymes [108], and possibly *NAMPT* [109]. Targeting microRNA, such as antagonizing hepatic miR-34a, has also been reported to increase *NAMPT* expression and NAD+ and SIRT1 activity *in vivo* [110].

## 5. Inhibition of NAD+ Consumers

Inhibiting the nonsirtuin NAD+ consumers also increases NAD+ levels and favors sirtuin activity. Inhibitors of PARPs or CD38 induce NAD+ levels, upregulate sirtuins, and enhance mitochondrial gene expression [67, 68, 89, 111]. PARP inhibitors are effective anticancer agents through DNA damage repair and improved oxidative metabolism (opposing the Warburg effect) in which the NAD+-sirtuin axis may be implicated [112–115]. The first PARP inhibitor, olaparib, is now licensed in the United States and Europe for the treatment of ovarian cancer [116, 117]. Therefore, PARP inhibitors may undergo further studies as NAD+-sparing agents to improve adaptive metabolism [118]. Interestingly, troxerutin and proanthocyanidins also inhibit PARPs in mice, thereby contributing to increased NAD+ [106, 108].

## 6. Type 2 Diabetes Mellitus

The global burden of obesity, insulin resistance, and type 2 diabetes mellitus (T2DM) continues to limit population health through increased cardiovascular disease risk and premature death [119].

Several studies support the notion that defective mitochondrial structure and function are strongly linked to insulin resistance and T2DM [120–128]. The most described mechanism is via defective mitochondrial fatty acid oxidation and the resultant accumulation of intracellular fatty acid metabolites and reactive oxygen species decreasing insulin sensitivity [129–133]. In addition, perturbed oxidative phosphorylation (OXPHOS) may be a direct cause of insulin resistance [134]. Supporting this, obesity reduces mitochondrial enzymatic activities [135, 136] and engenders metabolic inflexibility [137]; the inability to limit fatty oxidation and switch to carbohydrate oxidation in response to diet (and therefore insulin stimulation) [138–141].

Impaired NAD+-mediated sirtuin signaling is also implicated in insulin resistance and T2DM. In particular, defective SIRT1 activity is thought to be a factor in impaired insulin sensitivity [142–148]. This is endorsed by the finding that metformin acts through hepatic SIRT1 activation as part of its diabetes ameliorating effects [149]; results similarly observed with resveratrol [150].

Lifestyle manipulations, such as CR and exercise, can reverse insulin resistance and T2DM and share common mechanistic pathways of AMPK activation leading to elevated NAMPT-mediated NAD+ generation and SIRT1 activity to enhance mitochondrial function [46, 61, 151, 152]. Corroborating the link to NAD+, adipocyte-specific *NAMPT* deletion in mice decreased adiponectin production and resulted in severe multiorgan insulin resistance [92]. Aside from insulin sensitization, NAD+ and SIRT1 regulate glucose-stimulated insulin secretion in pancreatic *β* cells [153–155]. NAMPT inhibition and the lack of SIRT1 resulted in pancreatic *β* cell dysfunction [93, 156–159]. Interestingly, *SIRT1* regulates the key components of the circadian clock, *CLOCK* and *BMAL1* [160, 161], and when circadian misalignment is induced in mice, reduced hepatic *BMAL1* and *SIRT1* levels and insulin resistance ensue [150].

These lines of evidence suggest that an alternate strategy is to increase the level of NAD+ available to affected cells and tissues. Indeed, the NAD+ precursors used to enhance target tissue NAD+ availability have demonstrated efficacy to improve insulin sensitivity and reduce diabetic burden and associated metabolic derangements in preclinical models [33, 162].

NMN administration restored *β* cell glucose-stimulated insulin secretion and hepatic and muscle insulin sensitivity in mouse models of induced glucose intolerance [33, 92, 93]. Furthermore, Nam treatment in obese rats with T2DM promoted sirtuin-induced mitochondrial biogenesis and improved insulin sensitivity [82]. Similarly, NR supplementation attenuated HFD-induced obesity in mice, improved insulin sensitivity and glucose tolerance, and ameliorated the adverse lipid profile [54, 162]. Moreover, leucine supplementation in obese mice increased NAD+, mitochondrial biogenesis, insulin sensitivity, and lipid disposal [107].

Thus far, clinical data are limited to acipimox and resveratrol. Acipimox increased tissue insulin sensitivity in T2DM [79, 163–168] and improved *β* cell function when combined with dapagliflozin [76]. However, the results have been inconsistent at times. For instance, acipimox treatment in obese nondiabetic persons alleviated free fatty acids and fasting glucose with a trend toward reduced fasting insulin and homeostatic model assessment of insulin resistance [77], whereas van de Weijer *et al.* [78] did not report similar benefits in individuals with T2DM by using euglycemic hyperinsulinemic clamp studies. However, in the later study, this may have been related to the rebound increase in fatty acids after short-term acipimox administration [169]. Similarly, many describe that resveratrol decreases glucose and insulin levels in patients with impaired glucose tolerance and diabetes [95, 96, 170, 171], whereas others have not observed these findings [172]. The conflicting results among these studies may be explained by the heterogeneity in the selection of study population, dose and duration of treatment, and the methods of assessing insulin sensitivity.

## 7. Nonalcoholic Fatty Liver Disease

Nonalcoholic fatty liver disease (NAFLD) is the most common cause of liver disease in the Western world, encompassing the spectrum of liver diseases, including simple steatosis, nonalcoholic steatohepatitis, cirrhosis, liver failure, and hepatocellular carcinoma [173]. Hepatic lipid accumulation, which leads to cellular dysfunction, termed lipotoxicity, forms the basis for the development of NAFLD [174–176]. Consequently, a set of metabolic adaptations supervene, such as increased *β* oxidation. This adaptation induces metabolic inflexibility and drives the oxidative stress and mitochondrial dysfunction that are apparent in NAFLD [177–180].

Sufficient NAD+ levels are essential for adequate mitochondrial fatty acid oxidation [181, 182], and lipid caloric overload in mice reduces hepatic NAD+ levels and triggers lipotoxicity [183]. Zhou *et al.* [184] demonstrated that hepatic NAD+ levels decline with age in humans and rodents, which may contribute to NAFLD susceptibility during aging. Likewise, ample evidence suggests that impaired hepatic SIRT1 and SIRT3 signaling contributes to NAFLD [183, 185–188] and that *SIRT1* overexpression reverses hepatic steatosis [189, 190]. Stressing the significance of adequate hepatic NAD+ homeostasis, aberrant NAD+ metabolism is also implicated in alcoholic hepatic steatosis [191, 192] and hepatocellular carcinoma [193].

Several strategies targeting NAD+ metabolism to enhance sirtuin signaling have proved beneficial in the context of NAFLD. Nam and resveratrol protected hepatocytes *in vitro* against palmitate-induced endoplasmic reticulum stress [64, 194]. NR attenuated the severe mitochondrial dysfunction present in fatty liver of mice on HFD via NAD+-mediated sirtuin activation [54, 195]. Remarkably, NR was able to target many of the molecular aspects of NAFLD pathogenesis, including decreasing hepatic expression of inflammatory genes, blood tumor necrosis factor-*α* levels, and the hepatic infiltration by CD45 leukocytes [196]. PARP inhibition in mice with NAFLD can correct NAD+ deficiency, augmenting mitochondrial function and insulin sensitivity and allaying hepatic lipid accumulation and transaminitis [197]. Considering the current data, and in the absence of licensed therapies for NAFLD, replenishing the hepatic NAD+ pool to activate sirtuins and tackle mitochondrial dysfunction is staged for assessment in human clinical studies.

## 8. Aging and Metabolic Decline

By the year 2050, it is projected that the US population aged ≥65 years will be 83.7 million [198], with other low-mortality countries displaying similar population proportions [199].

Sarcopenia, Greek for “poverty of flesh,” is a consistent manifestation of aging, associated with frailty, metabolic disease, cardiovascular morbidity and mortality, and substantial health care costs [200, 201]. Needless to say, strategies aimed at treating sarcopenia and age-related diseases are needed.

A decline in NAD+ homeostasis contributes to the aging process [202, 203]. Indeed, NAD+ and sirtuins regulate diverse pathways that control aging and longevity [31, 57, 204–206], converging on the ability to defend mitochondrial function [207]. Certainly, mitochondrial dysfunction and defective cellular energy signaling have emerged as critical in aging and age-related metabolic diseases, such as T2DM, NAFLD, and sarcopenia [55]. Specifically, altered mitochondrial homeostasis, through reduced NAD+ and SIRT1 activity, is advocated as a hallmark of muscle aging [56]. In addition, limiting NAD+ in mouse skeletal muscle induced the loss of muscle mass and function (*i.e.,* sarcopenia) [208].

Age-related decline in NAD+ results from several mechanisms, which include accumulating DNA damage (and, consequently, chronic PARPs activation) [209, 210] and increased expression of CD38, clearing NAD+ and inducing mitochondrial dysfunction [211]. Additionally, chronic inflammation [212], a common feature in aging, reduces *NAMPT* expression and the ability to regenerate adequate NAD+ in multiple tissues [154].

The potential of NAD+ supplementation to support healthy aging is supported by several recent studies. *NAMPT* overexpression in aged mice matched the NAD+ levels and muscle phenotype of young mice [208]. Furthermore, *SIRT1* overexpressing mice were protected against the age-related development of diabetes and had a lower incidence of cancer [213]. NMN administration in aged mice restored NAD+ levels and the markers of mitochondrial function that decline with age [56].

Looking from a different angle, NR supplementation enhanced the expression of PGC-1*α* in the brain of a mouse model of Alzheimer’s disease, significantly attenuating the cognitive decline [214]. These findings affirm that decreased NAD+ levels contribute to the aging process and that NAD+ supplementation may prevent and even treat age-related diseases.

## 9. Discussion and Future Challenges

It is now well established that NAD+ is involved in metabolic regulation via redox and cell signaling reactions and that insufficient NAD+ is linked to a variety of metabolic and age-related diseases. The evidence reviewed here highlights that NAD+ levels can be therapeutically increased to potentiate sirtuins and mitochondrial function. This is a great opportunity in metabolic research that could conceivably lead to clinical utility.

The long-known lipid-lowering effects of NA may, at least partly, be NAD+ mediated. This hypothesis is favored because the half maximal effective concentration for the GPR109A receptor is in the nanomolar range [215, 216]; however, the therapeutic doses of NA are greatly in excess of this amount [71, 217]. Moreover, NR ameliorated hypercholesterolemia in mice without activating the GPR109A receptor [54]. Additionally, the liver lacks GPR109A receptors [218] but expresses liver X receptors, which regulate whole-body lipid homeostasis, that are upregulated by SIRT1 [219].

Although we have described the different pathways to NAD+ biosynthesis, it must be emphasized that not all tissues are capable of converting each precursor to NAD+ with equal efficacy, owing to the differences in the cell- and tissue-specific enzyme expression. For instance, cells must express the kynurenine pathway for *de novo* NAD+ synthesis, clearly active in the liver and brain [12], and must possess the Preiss–Handler pathway to use NA, which is active in most organs but less prominent in skeletal muscle. In contrast, the salvage pathways are crucial in all tissues to conserve NAD+ sufficiency [220]. Supporting this notion, the recommended daily allowance for NA is in milligrams, whereas an estimated 6 to 9 g of NAD+ are required daily to match turnover [58]. This is facilitated by the high affinity of NAMPT for Nam; thus, even small amounts of Nam are effectively converted to NMN and then NAD+ [221].

In the absence of head-to-head studies comparing the different compounds under defined conditions, it is currently not possible to identify the optimal NAD+ augmenting agent. The ubiquitous expression of *NRKs*, makes NR a precursor that can affect whole-body metabolism [162]. The inability of NR to activate the GPR109A receptor mitigates the undesirable NA side effects, and, unlike Nam, NR does not inhibit sirtuins. Furthermore, NAD+ generated from NR can target both nuclear and mitochondrial NAD+ pools, activating the respective compartmental sirtuins (*i.e.*, nuclear SIRT1 and mitochondrial SIRT3) [54]. This may be an advantage over other molecules, such as PARP inhibitors, with effects confined to the nucleus [67]. Similar to NR, NMN metabolism into NAD+ is governed by the salvage pathway. However, NMN availability has not been characterized in the diet [93, 222], unlike the naturally available NR.

In major proof-of-concept studies, therapeutically increasing NAD+ has been used to treat mouse models of mitochondrial diseases. Treatment of cytochrome C oxidase deficiency in mice with NR, PARP inhibition, and the AMPK agonist 5-aminoimidazole-4-carboxamide ribonucleotide reversed the mitochondrial dysfunction and improved muscle performance [223–225], effects attributed to NAD+ and sirtuins activation. Treatment of patients with T2DM by using acipimox resulted in improved skeletal muscle oxidative metabolism and mitochondrial function, measured by high-resolution respirometry [78]. However, this acipimox effect was not observed in obese persons without T2DM when assessed by phosphocreatine recovery magnetic resonance spectroscopy, mitochondrial biogenesis gene expression, and mitochondrial density on electron microscopy [77]. Two differences between these studies may explain the observed discrepancy. First, high-resolution respirometry is the current gold standard for *ex vivo* assessment of mitochondrial respiration if increased oxidative phosphorylation is the question [226]. Second, whereas mitochondrial dysfunction is evident in patients with T2DM, this is not prominent in obese persons without diabetes. Thus, the effects of NAD+ precursor supplementation may vary depending on the intervention and specific pathophysiologic conditions. Nam acts as an NAD+ precursor, increasing SIRT1 activity (below a threshold of sirtuin inhibition), or, conversely, a SIRT1 inhibitor, depending on the specific pathophysiologic state [84, 85].

We still have a limited understanding of the molecular interconversions of the administered NAD+ precursors. Illustrating this, administered NR is converted to Nam in the circulation before entering the cell [208, 227], whereas NMN is transformed extracellularly into NR, which then enters the cell and converts into NAD+ [227].

Knowledge gaps still persist in the role of sirtuins in different contexts. Some reports suggest that not all beneficial SIRT1 activation is through NAD+ and that cyclic adenosine monophosphate plays a role, independent of NAD+, in low-energy states [228, 229]. Upon pharmacologic NAMPT inhibition, Nam failed to increase NAD+; however, this did not prevent *SIRT1* upregulation, which was secondary to Nam-induced increase in intracellular cyclic adenosine monophosphate [64].

An important question is whether amplifying NAD+ and sirtuin activity is always desirable. SIRT1 upregulates T helper 17 cells that contribute to autoimmune disease when hyperactivated [230]. Correspondingly, SIRT1 inhibition supports the development of the regulatory T cells that protect against autoimmunity [231, 232]. Therefore, it is possible that SIRT1 activation places susceptible individuals at increased risk for autoimmune diseases. In the same way, whereas NR supplementation increased muscle stem cell number in aged mice, thereby enhancing mitochondrial function and muscle strength, it reduced the expression of cell senescence and apoptosis markers [233]; the state of senescence is important to protect against carcinogenesis [234]. Also, increased *NAMPT* expression is reported in some malignancies, calling into question whether increasing NAD+ might support aspects of the tumorigenic process [235].

Given the effect of the NAD+-sirtuin pathway on mitochondrial and metabolic homeostasis, novel supplementation strategies (*e.g.,* using NR or NMN) may be exploited to increase endogenous NAD+ availability in the treatment of metabolic and age-related diseases. This is the time for carefully designed human clinical studies to further examine these compounds before we can propose them as being useful nutraceuticals to counteract metabolic disease.
